# An Open-Label Trial of Memantine for Cognitive Impairment in Patients with Posttraumatic Stress Disorder

**DOI:** 10.1155/2015/934162

**Published:** 2015-05-12

**Authors:** Sriram Ramaswamy, Jayakrishna Madabushi, John Hunziker, Subhash C. Bhatia, Frederick Petty

**Affiliations:** ^1^Department of Mental Health and Behavioral Sciences, VA Nebraska-Western Iowa Health Care System, 4101 Woolworth Avenue, Omaha, NE 68105, USA; ^2^Department of Psychiatry, Creighton University School of Medicine, 3528 Dodge Street, Omaha, NE 68131, USA; ^3^Baptist Health System, 3201 4th Avenue South, Birmingham, AL 35222, USA; ^4^Mental Health Service, Orlando VA Medical Service, 5201 Raymond Street, Orlando, FL 32803, USA; ^5^Department of Psychiatry, University of Central Florida College of Medicine, 6850 Lake Nona Boulevard, Orlando, FL 32827, USA

## Abstract

*Background*. Studies using standard neuropsychological instruments have demonstrated memory deficits in patients with PTSD. We evaluated the efficacy and safety of the N-methyl-D-aspartate antagonist memantine in veterans with PTSD and cognitive impairment. *Methods*. Twenty-six veterans with PTSD and cognitive impairment received 16 weeks of memantine in an open-label fashion. Cognition was assessed using the Spatial Span, Logical Memory I, and Letter-Number Sequencing subtests of the Wechsler Memory Scale III and the Repeatable Battery for the Assessment of Neuropsychological Status (RBANS). RBANS measures attention, language, visuospatial skills, and immediate and delayed memories. The Clinician Administered PTSD Scale (CAPS), Hamilton Depression Scale (HAM-D), Hamilton Anxiety Scale (HAM-A), Quality of Life Enjoyment and Satisfaction Questionnaire (Q-LES-Q), and Sheehan Disability Scale (SDS) were secondary outcome measures. *Results*. There was a significant improvement in RBANS, both total and subscale scores (*P* < 0.05), over time. There was a reduction in total CAPS scores, avoidance/numbing symptoms (CAPS-C) and hyperarousal symptoms (CAPS-D), HAM-D, Q-LES-Q, and SDS scores. However, there was no reduction in reexperiencing (CAPS-B) and HAM-A scores. Memantine was well tolerated. *Conclusions.* Memantine improved cognitive symptoms, PTSD symptoms, and mood in veterans with PTSD. Randomized double-blind studies are needed to validate these preliminary observations.

## 1. Background

Posttraumatic stress disorder (PTSD) is a major public health problem with lifetime prevalence rate of up to 8% [1]. PTSD is more frequent after combat exposure and about 20–30% of Vietnam War veterans developed PTSD [2]. Prevalence among veterans returning from Iraq and Afghanistan is found to be as high as 17% [3] and is associated with debilitating psychological symptoms and poor quality of life.

In addition to amnesia for trauma-related stimuli, several studies have demonstrated cognitive deficits in different areas in individuals with PTSD. Deficits in verbal memory [4, 5], working memory and attention [6–10], processing speed [6], and nonverbal memory [11, 12] have been reported. A study reported short delay and long delay memory deficits in PTSD [13]. It may be argued that the comorbidities associated with PTSD could contribute to associated cognitive symptoms. However, even after controlling for confounding factors like head injury, depressive symptoms, and alcohol, significant cognitive impairment was reported in individuals with postwar PTSD [14, 15]. The neurobiology of cognitive impairment associated with PTSD has not been well elucidated. Available literature suggests involvement of the hypothalamic-pituitary-adrenal (HPA) axis leading to corticotrophin-releasing factor (CRF) dysregulation in PTSD. Variation in cortisol levels has been reported in PTSD, with most of the data suggesting hypocortisolemia as a long-term effect [16]. Some data suggest that acute stress induces elevations in endogenous corticosteroids, which may cause hippocampal damage, which in turn might be associated with memory impairment in PTSD [17, 18]. The glutamatergic system also plays a role in stress response, which is mediated by cortisol dysregulation. Glutamate and CRF appear to modulate each other's expression. Rats exposed to immobilization stress show increased expression of the N-methyl-D-aspartate (NMDA) receptor subunit in the paraventricular nucleus in the hypothalamus [19, 20]. Open-label trials of lamotrigine, an antiglutamatergic medication, in PTSD support the role of glutamatergic abnormalities in PTSD [21].

Currently, the only FDA approved medications available for the treatment of PTSD are sertraline (Zoloft) and paroxetine (Paxil). Unfortunately, many patients with PTSD are unresponsive, have only moderate or marginal responses, or have troubling side effects of first-line selective serotonin reuptake inhibitor (SSRI) treatment. As a result, there has been considerable interest in alternative pharmacological treatments for PTSD, including medications to target cognitive symptoms of PTSD. Memantine is an NMDA glutamate receptor antagonist that is approved by the US Food and Drug Administration (FDA) for treatment of dementia, and it has also been used off-label for a variety of psychiatric disorders such as major depression, bipolar disorder, schizophrenia, and anxiety disorders (for reviews, see [22–24]). While there have been no controlled studies examining the effects of memantine on PTSD, there is evidence from animal research that memantine can reduce anxiety and improve cognition [25], and case studies in humans suggest that memantine may help to treat cognitive symptoms in patients with combat-related PTSD [26, 27]. Further study is needed, however, to establish the safety and efficacy of memantine in this population. We conducted a prospective open-label study to test the hypothesis that memantine's antiglutamatergic activity could improve cognitive functioning and overall symptoms of PTSD. The secondary aim of this study was to find out usefulness of memantine for core PTSD symptoms and comorbid depressive symptoms.

## 2. Design and Methodology

Participants were recruited from the Omaha Veterans Affairs Medical Center following local IRB approval. We obtained written consent from all the subjects who were recruited in the study. Twenty-six veterans (25 males, 1 female) between the ages of 19 and 65 years (M = 56.4; SD = 4.5) with chronic PTSD (diagnosis for >6 months) attributable to military combat exposure were included in the study. Patients were required to be clinically stable on their psychotropic medication regimen for at least three months prior to study entry. In addition to meeting DSM-IV criteria for PTSD and endorsing subjective complaints of memory difficulties, patients had to score at least one standard deviation below the mean performance of a standardized, age- and sex-matched population on the Spatial Span, Logical Memory I, and Letter-Number Sequencing subtests of the Wechsler Memory Scale III (third edition) for study entry. Patients with a history of dementia, schizophrenia, bipolar disorder, traumatic brain injury, and seizure were excluded. Patients with any history of alcohol or illicit drug abuse or dependence within the past one month were excluded. Patients requiring concomitant treatment with drugs with potential effects on the glutamatergic system, such as amantadine, dextromethorphan, or carbonic anhydrase inhibitors, were excluded.

Subjects initially received memantine of 5 mg once daily, which was increased weekly by 5 mg/day in divided doses to a dose of 20 mg/day. Memory was assessed using the Repeatable Battery for the Assessment of Neuropsychological Status (RBANS) [28] using both forms A and B at baseline, end of week 8, and end of week 16. The RBANS is composed of 10 subtests that yield a total score and five index scores: immediate memory, visuospatial/constructional, language, attention, and delayed memory. Each index score has a normal mean of 100 and standard deviation of 15 based on the performance of a standardization sample matched to the U.S. Census on sex, ethnicity, and level of education. Alternative forms of the RBANS (forms A and B) were used to avoid bias due to practice effects. We administered Clinician Administered PTSD Scale (CAPS), Hamilton Depression Scale (HAM-D), Hamilton Anxiety Scale (HAM-A), Quality of Life Enjoyment and Satisfaction Questionnaire (Q-LES-Q), and Sheehan Disability Scale (SDS) to assess the secondary measures. Changes in the scores over time with repeated measures were estimated with mixed-effects models. The primary outcome measures of interest were index and percentile scores in RBANS total and subscale scores. The repeated measures model included visit (as a categorical variable) as a fixed effect. An unstructured covariance matrix was used to fit the within patient repeated measures effect. Tukey's method was used to compare pairwise means.

Secondary outcome measures, CAPS, HAM-A, HAM-D, Q-LES-Q, and SDS, were analyzed similarly to the RBANS with repeated measures models. *P* values less than 0.05 are considered to be statistically significant. SAS software version 9.1 (SAS Institute, Cary, NC) was used for the analysis.

## 3. Results

**Figure 1:**
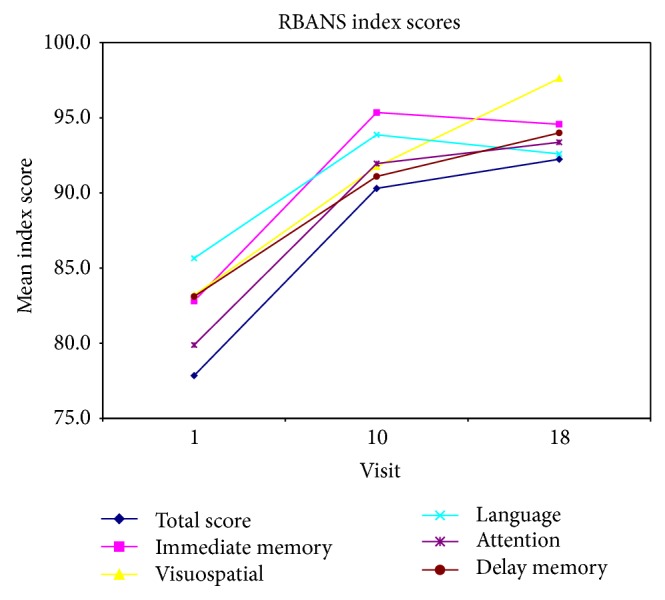
This figure illustrates changes in Repeatable Battery for the Assessment of Neuropsychological Status index scores over time.

**Figure 2:**
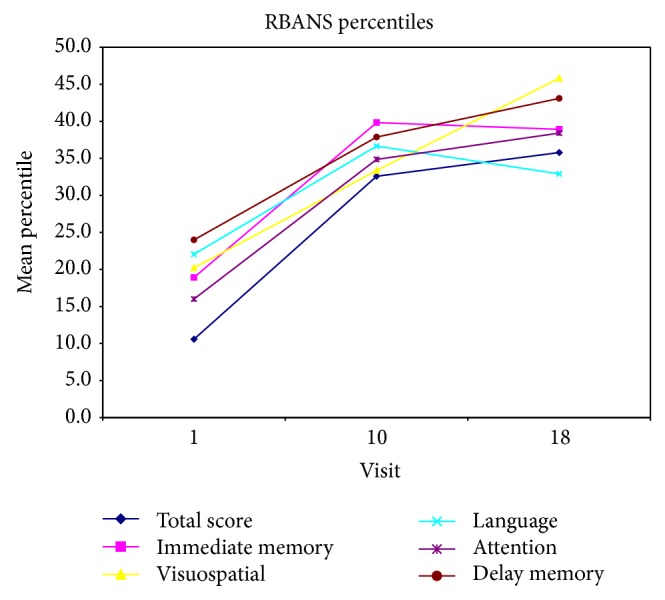
This figure illustrates changes in Repeatable Battery for the Assessment of Neuropsychological Status percentile scores over time.

A total of 26 patients were included in the study. Participants were compliant with their appointments; only three patient visits during the entire study were missed. Significant change was found in RBANS total and subscale scores during the follow-ups at baseline, end of week 8, and end of week 16 (Table 1). For RBANS, the total score and subscales all changed significantly over time, whether looking at index scores or percentiles (all *P* < 0.01). Figures 1 and 2 illustrates changes in RBANS index scores and percentile scores respectively over time.  Table 3 illustrates the estimated means and standard errors for secondary outcome scores at each visit. Age and gender were not contributing factors to the results. This is not surprising since almost all subjects were males and the age ranges very small.

## 4. Secondary Outcome Measures

Secondary outcomes for this study included CAPS, HAM-A, HAM-D, Q-LES-Q, and SDS.  Table 2 shows the results of the repeated measures, mixed-effects (ME) model for secondary outcomes. CAPS-C, CAPS-D, CAPS total, HAM-D, Q-LES-Q, and SDS all changed significantly over time (all *P* < 0.05). CAPS-B and HAM-A showed no overall change over time.

**Figure 3:**
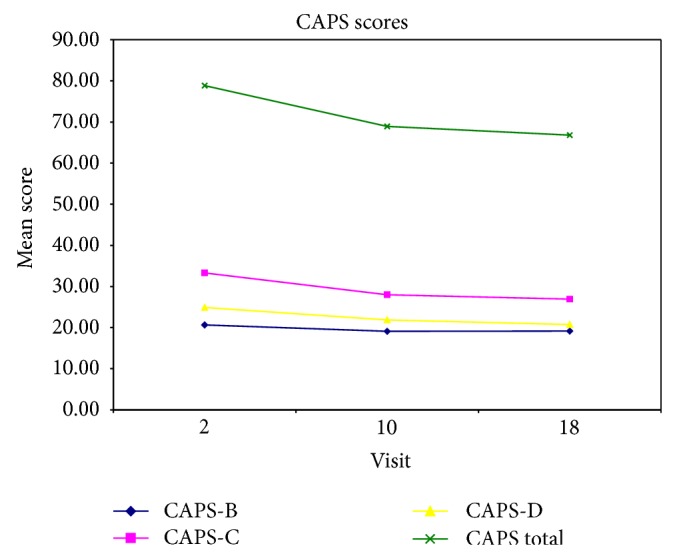
This figure illustrates changes in Clinician Administered PTSD Scale scores over time.

**Figure 4:**
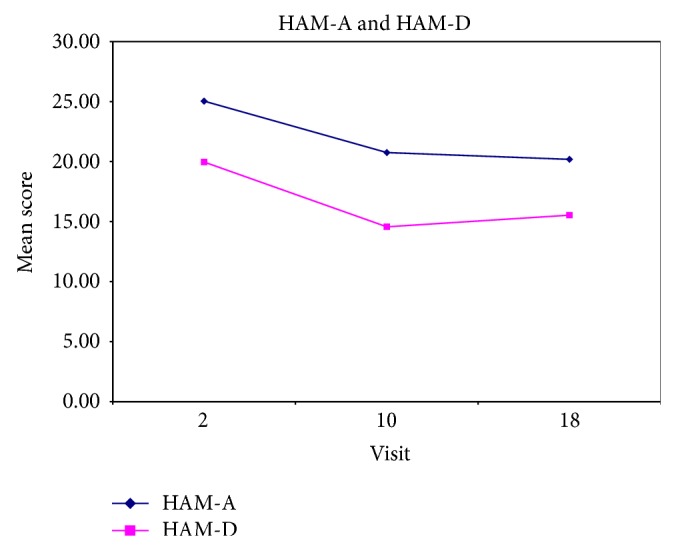
This figure illustrates changes in Hamilton Depression Scale and Hamilton Anxiety Scale scores over time.

**Figure 5:**
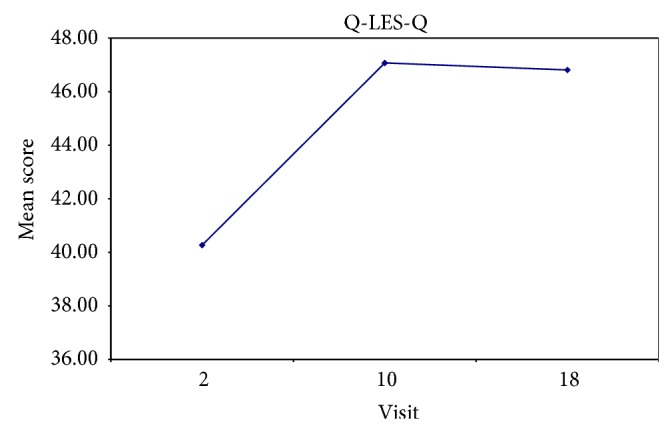
This figure illustrates changes in Quality of Life Enjoyment and Satisfaction Questionnaire scores over time.

Adverse effects were mild in nature. There were no serious adverse effects in the trial. Two subjects were lost to follow-up after completing the baseline visit. One subject experienced constipation, so the drug was not increased above 10 mg in that particular subject. Figures 3, 4, and 5 illustrate changes in CAPS, HAM-A, HAM-D scores over time.  Figure 5 illustrates changes in Quality of Life Enjoyment and Satisfaction Questionnaire scores over time.

## 5. Discussion

Chronic PTSD is associated with cognitive dysfunction [4, 6, 11]. Dysregulations in the hypothalamic-pituitary-adrenal (HPA) axis, cortisol system, and glutamate pathways are associated with PTSD symptoms. Limited data is available regarding the neurobiology of cognitive symptom in PTSD. Glutamate and its subreceptors are intrinsically linked to memory process by their involvement in long-term potentiation (LTP) and long-term desensitization (LTD), mechanisms underlying learning and memory. However, the role of the glutamatergic system in cognitive dysfunction associated with PTSD is not well elucidated. It has been shown that stress can affect the glutamatergic system, presumably through the cortisol pathway [21]. There is a dearth of studies looking at pharmacological interventions for cognitive symptoms of PTSD.

Memantine, which has actions on the glutamatergic system, has been approved by the FDA for dementia. In a pilot study [26], memantine was reported to be useful for delayed recall measure of memory, variable reduction of depressive symptoms, and variable reduction in hyperarousal symptoms. Results of our study demonstrate improvement in cognitive symptoms in PTSD patients following therapy with memantine for 16 weeks. Improvement in memory was observed as early as 8 weeks, reflected by improvement in RBANS total score and subscales over time in both index scores and percentiles.

This is consistent with the results in Alzheimer's disease studies, where differences between memantine and placebo in cognition were observed after 8 weeks [29]. Except for the visuospatial percentile scores, the remainder of our scores demonstrated improvement beyond 8 weeks. There was an increase in the proportion of subjects who attained normal scores of attention, visuospatial skills, and immediate and delayed memories with memantine at subsequent visits compared to the initial visit. In our study memantine was associated with significant improvement across all RBANS subscales. This is in contrast to several negative studies of memantine in cognitive dysfunction associated with schizophrenia. The neurobiology of both PTSD and schizophrenia cognitive impairment is not fully known although there is stronger evidence for cholinergic dysfunction in schizophrenia than in PTSD. The robust effect in our study might be explained by a strong placebo response, a task learning effect, or merely confirmatory of glutamatergic abnormalities in chronic PTSD. Nevertheless, the results were surprising and support investigation with larger controlled trials.

Improvement was also noticed in PTSD symptoms, including hyperarousal, avoidance, and depressive symptoms, as demonstrated by improvement in CAPS scores and HAM-D scores. Memory disturbances are predominant in the presentation of posttraumatic stress disorder (PTSD) and are part of the diagnostic criteria. There was no improvement in the reexperiencing set of symptoms (CAPS-B). The reexperiencing symptom criteria of PTSD include intrusive memories of the traumatic event. It is plausible that a true effect of memantine on this subset of PTSD symptoms might have been missed secondary to its effect on improving overall declarative memory.

To summarize, we observed improvement in memory, core symptoms of PTSD, and depression in combat veterans with PTSD following open-label treatment with memantine. Our study was limited by the nature of its open-label, nonplacebo controlled study design and small sample size. The open-label design was chosen for its advantages for a proof of concept study such as simplicity, ease of patient recruitment and retention. Memory performance has been associated with education on the RBANS. [30] Also, the relationship between chronicity of PTSD and cognitive decline has to be borne in mind especially since there is data that prolonged PTSD that may have cumulative adverse effects on hippocampal volume [31]. Our study did not look at the effect of education and chronicity of PTSD on the outcome variables. Future studies should control for these important variables. Despite the limitations our data are the best currently available and provide useful insights into the management of cognitive impairment associated with PTSD. Larger, double-blind, placebo-controlled, randomized, and controlled trials are warranted to validate the findings of this study.

## Figures and Tables

**Table 1:** Demographic and baseline characteristics.

	*N* (%)
Ethnicity	
African American	3
White	23 (76)
Hispanic	0
Asian	0
Other	0
Gender	
Male	24 (92)
Female	2 (8)
Age (mean/SD)	56.88 years (3.9)

**Table 2:** Change in RBANS scores during repeated measures.

	Visit	Mean	SE	*P* value
Total score	Baseline	77.8	2.0	<0.0001
	Week 8	90.3	2.6	
	Week 16	92.2	2.7	

Total score percentile	Baseline	10.6	1.6	<0.0001
	Week 8	32.6	4.9	
	Week 16	35.8	5.4	

Immediate memory	Baseline	82.8	2.5	<0.0001
	Week 8	95.3	2.3	
	Week 16	94.6	2.7	

Immediate memory percentile	Baseline	18.9	4.4	<0.0001
	Week 8	39.8	5.3	
	Week 16	38.9	5.8	

Visuospatial index score	Baseline	83.2	2.6	0.0001
	Week 8	91.8	2.9	
	Week 16	97.6	3.1	

Visuospatial percentile	Baseline	20.2	3.8	0.0002
	Week 8	33.4	5.6	
	Week 16	45.8	5.9	

Language	Baseline	85.7	2.2	0.0024
	Week 8	93.9	1.9	
	Week 16	92.6	1.7	

Language percentile	Baseline	22.0	3.7	0.0006
	Week 8	36.7	3.5	
	Week 16	32.9	3.7	

Attention	Baseline	79.9	2.7	0.0017
	Week 8	92.0	3.0	
	Week 16	93.4	3.3	

Attention percentile	Baseline	16.0	3.1	0.0009
	Week 8	34.9	5.9	
	Week 16	38.4	6.3	

Delayed memory	Baseline	83.1	3.5	0.0003
	Week 8	91.1	3.6	
	Week 16	94.0	3.5	

Delayed memory percentile	Baseline	24.0	4.4	0.0003
	Week 8	37.9	5.6	
	Week 16	43.1	5.5	

There was significant improvement in all RBANS scores, including language, attention, and immediate and delayed memory from baseline, week 8, and week 16.

**Table 3:** Estimated means and standard errors for secondary outcome scores at each visit (from ME model).

	Week	Mean	SE	*P* value
CAPS-B	Baseline	20.65	1.73	0.52
	End of week 8	19.10	1.62	
	End of week 16	19.17	1.60	

CAPS-C	Baseline	33.31	1.54	0.0055
	End of week 8	27.98	2.08	
	End of week 16	26.92	1.97	

CAPS-D	Baseline	24.88	0.97	0.023
	End of week 8	21.88	1.58	
	End of week 16	20.75	1.44	

CAPS total score	Baseline	78.85	3.79	0.027
	End of week 8	68.92	4.72	
	End of week 16	66.80	4.45	

HAM-A	Baseline	25.04	1.71	0.056
	End of week 8	20.76	1.99	
	End of week 16	20.18	1.74	

HAM-D	Baseline	19.96	1.17	0.013
	End of week 8	14.57	1.36	
	End of week 16	15.53	1.54	

Q-LES-Q	Baseline	40.27	1.79	0.0076
	End of week 8	47.07	2.25	
	End of week 16	46.81	2.21	

SDS	Baseline	7.02	0.31	0.020
	End of week 8	6.20	0.66	
	End of week 16	5.73	0.50	
